# Sodium Ascorbate induces apoptosis in neuroblastoma cell lines by interfering with iron uptake

**DOI:** 10.1186/1476-4598-6-55

**Published:** 2007-08-30

**Authors:** Roberta Carosio, Guendalina Zuccari, Isabella Orienti, Salvatore Mangraviti, Paolo G Montaldo

**Affiliations:** 1 Laboratory of Oncology, G. Gaslini Institute, Genova, Italy; 2 Department of Pharmaceutical Sciences, University of Bologna, Bologna, Italy; 3 Clinical Pathology Laboratory, G. Gaslini Institute, Genova, Italy

## Abstract

**Background:**

Neuroblastoma (NB) is an extra-cranial solid tumour of childhood. In spite of the good clinical response to first-line therapy, complete eradication of NB cells is rarely achieved. Thus, new therapeutic strategies are needed to eradicate surviving NB cells and prevent relapse. Sodium ascorbate has been recently reported to induce apoptosis of B16 melanoma cells through down-regulation of the transferrin receptor, CD71. Since NB and melanoma share the same embryologic neuroectodermal origin, we used different human NB cell lines to assess whether the same findings occurred.

**Results:**

We could observe dose- and time-dependent induction of apoptosis in all NB cell lines. Sodium ascorbate decreased the expression of CD71 and caused cell death within 24 h. An increase in the global and specific caspase activity took place, as well as an early loss of the mitochondrial transmembrane potential. Moreover, intracellular iron was significantly decreased after exposure to sodium ascorbate. Apoptotic markers were reverted when the cells were pretreated with the iron donor ferric ammonium citrate (FAC), further confirming that iron depletion is responsible for the ascorbate-induced cell death in NB cells.

**Conclusion:**

Sodium ascorbate is highly toxic to neuroblastoma cell lines and the specific mechanism of vitamin C-induced apoptosis is due to a perturbation of intracellular iron levels ensuing TfR-downregulation.

## Background

Neuroblastoma is the most common solid extracranial tumor of childhood [1]. This tumor has long fascinated clinicians and biologists due to its enigmatic behaviour. Extreme clinical heterogeneity is seen, including spontaneous regression. Still, a large part of neuroblastoma patients have highly aggressive disease that is refractory to intensive therapies and ultimately fatal. Current therapy for high risk neuroblastoma has reached a near-maximally tolerable state that includes cytoreductive and myeloablative therapies, radiation, autologous or allogenic bone marrow transplant, retinoids and immunomodulators, among others [2].

Therefore, less toxic and more effective therapy are to be found.

It has been recently reported that vitamin C is effective in a large panel of tumor cell lines [3,4]. Sodium Ascorbate (vitamin C) has a controversial history in cancer treatment. The discrepancy obtained in research and analysis by many authors was principally due to the different dose and route of administration of vitamin C and consequently to its plasma concentration [5-7].

Given this situation, the role of vitamin C in cancer treatment has been reexamined; in fact apparent responses of malignant disease to intravenous ascorbate therapy have appeared as case reports [5,6,8]. Some *in vitro *studies showed that ascorbate causes toxicity to cancer cells at concentration that do not affect normal cells [3,9,10]; furthermore, it was reported that melanoma cells were more susceptible to ascorbate toxicity than any other tumor cells. Ascorbate seems to induce apoptosis by inducing disequilibrium of iron uptake due to down-regulation of transferrin receptor [4]. Since melanoma and neuroblastoma have the same embryological neuroectodermal origin, we investigated whether neuroblastoma cell lines have the same susceptibility to vitamin C. First we studied the expression of transferrin receptor in neuroblastoma cell lines. Neoplastic cells were reported to express high rate of transferrin receptor, because growing cells have an increased requirement of iron [11]. Neuroblastoma cells are not an exception to this general rule, to the point that iron chelators have been proposed for the treatment of this kind of tumor [12,13].

## Results

### Effect of Sodium Ascorbate on neuroblastoma cell lines

**Figure 1:**
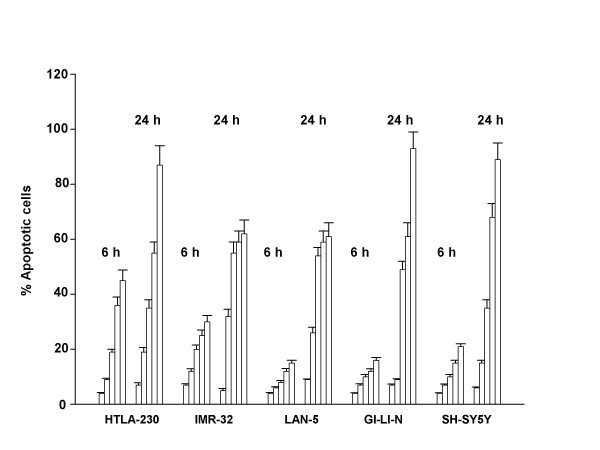
Sodium ascorbate induces apoptosis in neuroblastoma cell lines. Cells were seeded in six well plates and treated for 6 or 24 hours with increasing concentrations of sodium ascorbate. For each bar group, the concentration was: 0 mM (control), 0.5 mM, 1 mM, 2 mM, 3 mM. Cells were then processed and stained with propidium iodide as described in "Materials and Methods". The percent of cells in the sub-G_1 _phase of the cell cycle were considered apoptotic. The data are the mean ± S.D. from three independent experiments.

We first investigated whether sodium ascorbate had cytotoxic effects on neuroblastoma cell lines. Therefore we used sodium ascorbate at millimolar concentration ranging from 0.5 mM to 3 mM for 24 hours. Vitamin C concentrations causing a 50% decrease in cell survival after 24 hours (EC_50 _values, table 1) were less than 2 mM for all five cell lines, as assessed by trypan blue exclusion method. Morphological inspection of treated cells, suggested that cell death took place through apoptosis rather than necrosis. This was confirmed by cytofluorimetric analysis of DNA content. There was a dose- and time-dependent increase of cells in the sub-G_1 _phase of the cell cycle (typical of apoptotic cells) upon treatment with sodium ascorbate (fig. 1).

**Table 1:** EC_50 _values of vitamin C in human neuroblastoma cell lines. Cells were treated with sodium ascorbate for 24 h, EC_50 _values were determined by using trypan blue exclusion method. Values indicated are the mean ± S.D. of three separate experiments.

*Neuroblastoma cell lines*	*EC_50 _(mM) 24 h*
HTLA-230	1.78 ± 0.3
IMR-32	0.89 ± 0.04
LAN-5	0.88 ± 0.03
GI-LI-N	0.98 ± 0.04
SH-SY5Y	1.44 ± 0.2

### Sodium ascorbate induced-down regulation of TfR expression

**Figure 2:**
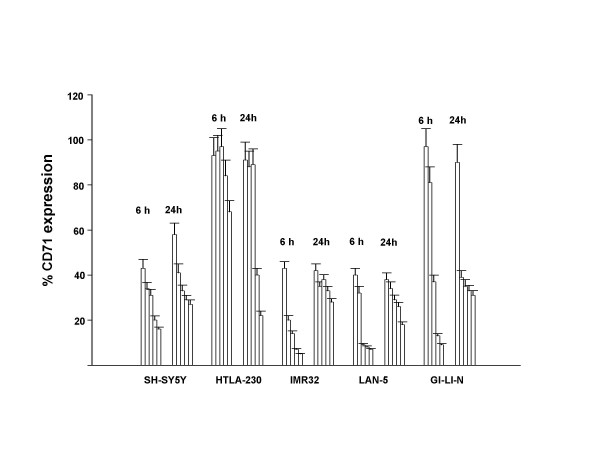
Membrane expression of TfR (CD71) by neuroblastoma cell lines and its modulation by sodium ascorbate. Cells were seeded in six well plates and treated for 6 and 24 hours with increasing concentrations of sodium ascorbate. For each bar group, the concentration was: 0 (control), 0.5 mM, 1 mM, 2 mM and 3 mM. Cells were than washed and incubated with a FITC-coniugated mouse monoclonal antibody specific to human CD71 and analyzed by flow cytometry. The data are the mean ± S.D. from three independent experiments, each in triplicate.

Many tumor cells display high level of transferrin receptor to meet the increase in iron supply required by growing cells [11] and several authors have reported that antibodies against TfR have antiproliferative effects in vitro and in vivo [14,15]; furthermore, it was reported that in melanoma cells ascorbate induced down-regulation of cell surface TfR [4]. In the present work, all the neuroblastoma cells analyzed expressed TfR (fig. 2). Addiction of sodium ascorbate resulted in a concentration dependent down-regulation of TfR; besides, TfR change of expression seemed to be time independent, since there was a substantial decrease of CD71 expression at 6 hours and did not significantly decrease at 24 hours (fig. 2).

### Intracellular iron level

**Figure 3:**
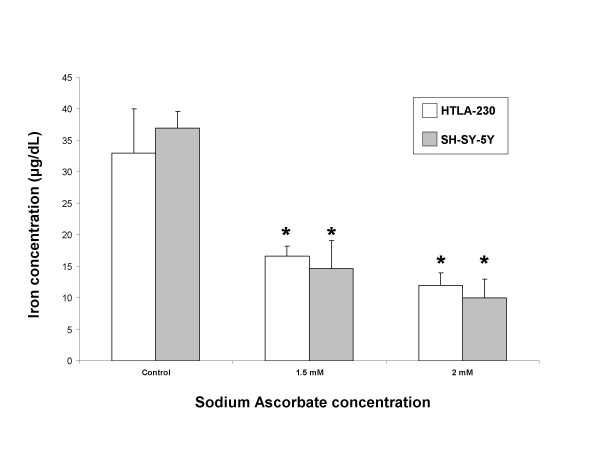
**Reduction of intracellular iron levels by sodium ascorbate**. 30 × 10^6 ^of either HTLA-230 or SH-SY5Y were treated with the indicated doses of sodium ascorbate for 24 hours and iron levels were measured in cell lysates. After terminating the incubation, cells were collected by scraping and washed three times with PBS and then lysed in specific buffer. Iron levels were analyzed with a Cobas Integra 800 system as described under "Material and Method". Values indicated are the mean ± S.D. of three separate experiments.

In order to find the possible mechanism of neuroblastoma cells apoptosis induced by vitamin C, we investigated whether this phenomenon was correlated with change in the intracellular iron levels; therefore we treated HTLA-230 and SH-SY5Y for 24 hours with 1.5, and 2 mM sodium ascorbate and iron level was then measured. As shown in fig. 3 vitamin C significantly reduced intracellular iron levels in a dose-dependent manner.

### Effect of vitamin C on mitochondrial membrane potential

**Figure 4:**
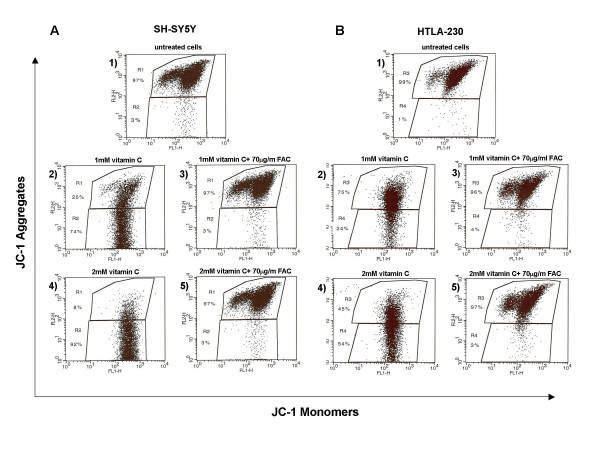
Representative example of cytofluorimetric analysis showing the effect of vitamin C alone or in combination with FAC, on the mitochondrial membrane potential of SH-SY5Y (panel A) and HTLA-230 (panel B) stained with JC-1 probe. In abscissa FL-1 (green fluorescence); in ordinate FL-2 (red fluorescence). Numbers represent the percentage of non-apoptotic red fluorescent cells (R1, R3) and that of apoptotic green fluorescent cells (R2, R4). Sodium ascorbate induces a marked increase in apoptosis which is fully prevented by treatment with FAC. SH-SY5Y and HTLA-230 were incubate in complete medium in the absence (A1, B1) or in presence of 1 mM sodium ascorbate (A2, B2) and 2 mM sodium ascorbate (A4, B4) for 16 hours. Cells were pretreated (A3, A5, B3, B5) with 70 μg/ml of FAC for 3 hours. Comparable results were obtained in three independent experiments.

It is well established that the reduction of Δψ_m _also plays a key role in triggering apoptosis [16]. Treatment with vitamin C induced depolarization of the mitochondrial transmembrane potential in all neuroblastoma cell lines investigated (fig. 4, panel A2, A4, panel B2, B4).

To confirm that iron is involved in the induction of apoptosis we tested whether the iron donor ferric ammonium citrate (FAC) was able to prevent the sodium ascorbate-induced apoptosis. To this aim before incubation with 2 mM of vitamin C, we treated HTLA-230 cells with 70 μg/ml of FAC for 3 hours; afterwards we assessed the induction of apoptosis by phosphatidylserine externalization using FITC-Annexin V binding.

**Figure 5:**
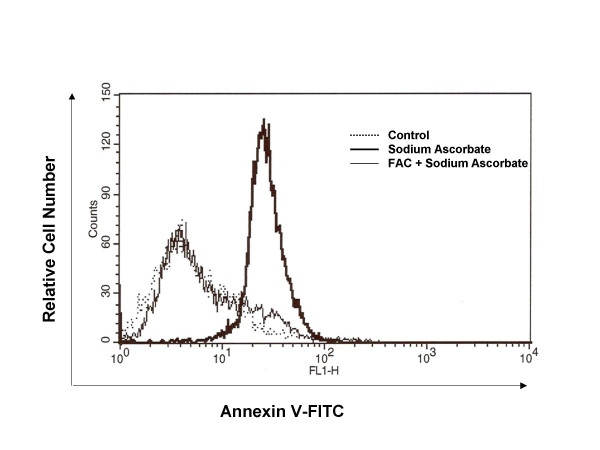
**Effect of FAC on sodium ascorbate-induced apoptosis**. HTLA-230 were grown in either complete medium alone (control cells), or medium containing 2 mM of sodium ascorbate (in the presence or absence of FAC 70 μg/ml). After 16 hours, cells detached in the medium were collected by centrifugation, resuspended and incubated with AnnexinV-FITC. Apoptosis was quantified as increased green fluorescence by flow cytometry. The data are the mean ± S.D. from four independent experiments. Statistical analysis was done by Student's test (*, p < 0.005).

The data depicted in fig. 5 show that the peak of apoptosis induced by ascorbate is nearly abolished by the pretreatment with the donor of iron FAC; in fact the percentage of Annexin V-positive cells decreased from 82% (cells incubated only with ascorbate) to 15% (cells incubated with ascorbate and pretreated with FAC). Similar results were obtained when we analyzed changes in the mitochondrial membrane potential (Δψ_m_) in HTLA- 230 and SH-SY-5Y, measured as the ratio of red-to-green JC-1 fluorescence (fig. 4). Treatment with vitamin C induced depolarization of the mitochondrial transmembrane potential in all neuroblastoma cell lines investigated and the effect was inhibited by FAC.

### Sodium Ascorbate-induced apoptosis is mediated by caspases activation

**Figure 6:**
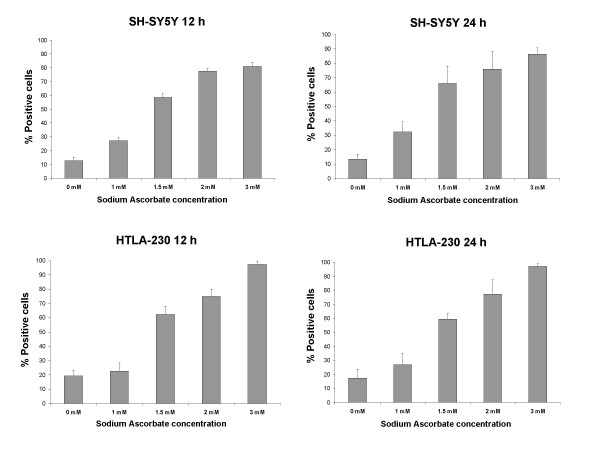
Effect of vitamin C on caspases activation. HTLA-230 and SH-SY5Y were treated with the indicated concentrations for 12 and 24 hours. After detachment, cells were stained with the pan-caspase-fluorescent-probe z-VAD-FMK according to the manufactures instructions. Relative increases in fluorescence emission were detected by single color flow cytometry. The data are the mean ± S.D. from three independent experiments.

**Figure 7:**
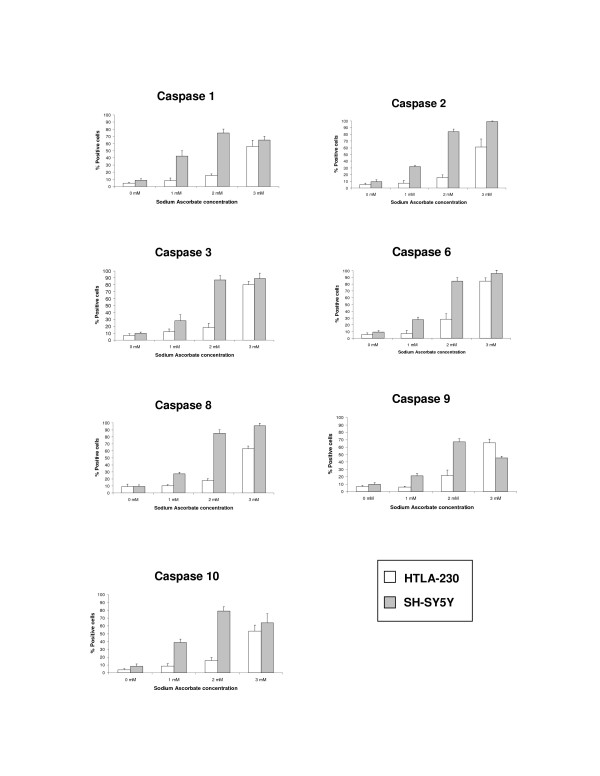
Effect of sodium ascorbate on single caspases activation. HTLA-230 and SH-SY5Y were incubated in the absence (0 mM) or presence of the indicated concentrations for 16 hours and then analyzed for single caspases by flow cytometry as described in Material and Method. Coloums, mean of three different experiments, each done in triplicate; bars, SD.

Apoptosis arises from proteolytic activation of the cysteine proteases called caspases [17,18]. To verify the involvement of caspases in sodium ascorbate-induced apoptosis in neuroblastoma cell lines, we used a fluorescence-based pan-caspase activity assay kit. Cells were treated with increasing concentrations of sodium ascorbate for 12 and 24 hours before incubation with the fluorescent probe. The results showed a dose-dependent shift in fluorescence in all tested cell lines, revealing a significant participation of caspases in ascorbate-induced apoptosis (in fig. 6 are represented only SH-SY5Y and HTLA-230, similar data were obtained for GI-LI-N, IMR-32 and LAN-5, data not shown). Moreover the activation of caspases was an early event; in fact there was not significant difference in fluorescence increase between 12 and 24 hours of incubation. To understand which caspases was mainly involved in the apoptotic mechanism, we used all the commercially available kits for single caspases (i.e., caspase-1, caspase-2, caspase-3&7, caspase-6, caspase-8, caspase-9, and caspase-10) and measured the increase in fluorescence after 16 hours in SH-SY5Y and HTLA-230 cell lines. As depicted in fig. 7, all the above caspases showed a similar pattern of activation, in particular in SH-SY5Y caspases were already activated at 1 mM ascorbate. Although these experiments did not allow us to individuate a hierarchy of activation, they clearly confirmed the apoptotic nature of cell death induced by sodium ascorbate in neuroblastoma cell lines.

## Discussion

The effect of vitamin C on cancer has been a subject of great controversy, and vast literature exists on this topic. Thirty years ago Cameron, Campbell and Pauling reported that high-dose vitamin C had beneficial effect for patient with terminal cancer. Subsequently, double-blind, randomized clinical trials, conducted by Moertel of the Mayo clinic, failed to show any benefit and the use of sodium ascorbate in cancer treatment was abandoned [19-24]. However, Moertel's results were not comparable to those of Cameron, as ascorbate was given orally and not intravenously. It was not recognized that the route of administration might produce large difference in plasma concentration. Recent clinical data show that when given i.v., ascorbate plasma concentration is 25 fold higher respect that of the same oral dose [7].

Sodium ascorbate produces cytotoxic effect in an array of malignant cell lines, including melanoma cells which are particularly susceptible [3,4,9,10].

In light of these results we investigated the effect of sodium ascorbate against neuroblastoma.

Our data show that sodium ascorbate killed neuroblastoma cells, using lower concentrations compared to those active in other tumor cell lines; in fact vitamin C, in the range of 0.5 to 3 mM turned out to be strongly cytotoxic. To broadly cover the phenotype of the tumor we used five neuroblastoma cell line including MYCN amplified (HTLA-230 and IMR-32) and not-MYCN amplified (SH-SY5Y). Sodium ascorbate-induced cell death was apoptosis, as documented by our experiments regarding the decrease of mitochondrial membrane potential, the phosphatidylserine externalization, the increase of cells in the sub-G_1 _phase of the cell cycle and caspases activation.

In this study we show that all the caspases are activated by the incubation with vitamin C, in fact, not only caspases related to the mitochondrial pathway were found to be activated but also those related to the death receptor pathway. The two pathways, extrinsic and intrinsic, are probably interconnected by caspase-8 mediated cleavage of the pro-apoptotic Bcl-2 family member Bid, producing a truncated Bid (tBid) fragment that promotes the mitochondrial release of proteins from the intermembrane space. In particular, it is known, that the release of cytochrome c induces the activation of caspase proteases through the induction of apoptosome formation [25].

To clarify if ascorbate-mediated cytotoxicity was due to perturbation in the iron uptake, we measured intracellular iron level of HTLA-230 and SH-SY5Y after 24 hours of incubation with increasing doses of vitamin C. We could observe a dose-dependent decrease of iron levels; furthermore, pretreatment of cells with the iron donor FAC completely prevented sodium ascorbate-induced apoptosis. Thus, the percentage of Annexin V positive cells decreased to that of untreated cells and, in a similar way, the pretreatment with FAC completely abolished the ascorbate-induced reduction of mitochondrial membrane potential.

Several studies report the correlation of iron level with apoptosis [26,27]. Virtually all living cells have an absolute requirement for iron, since many Fe-containing proteins catalyze key reactions involved in energy metabolism (cytochromes, mitochondrial aconitase, Fe-S proteins of the electron transport chain), respiration (hemoglobin), and DNA synthesis (ribonucleotide reductase) [11]. Inhibition of ribonucleotide reductase has been proposed as the cause of the growth arrest that occurs in cell deprived of iron because this enzyme requires iron to reduce ribonucleotides to form the substrate of DNA synthesis [28-30]. The regulated uptake and availability of iron is closely linked to cellular proliferation. Three proteins, the iron-transport transferrin, the transferrin receptor on the cell surface and ferritin are essential for making iron available for cellular use. Many neuroblastomas produce very large amount of ferritin [12]. In addition to being incorporated into heme proteins and enzymes, iron may participate more directly in the regulation of cell growth and apoptosis of neuroblastoma; in fact neuroblastoma cells appear to be unusually sensitive to the cytotoxic and cytostatic effect of iron chelators [31]. Furthermore the mechanism by which vitamin C decreased intracellular iron level seems to be correlated with the down-regulation of transferrin receptor. Our data are consistent with the fact that tumor cells express high level of transferrin receptor to meet the increase in iron required by growing tumor tissue [11].

Some *in vitro *studies showed that ascorbate causes toxicity to cancer cells at concentrations that do not affect normal cells [3,9]; this phenomenon is probably due to a tumor specific intracellular transport of ascorbate. Extracellular ascorbate is oxidized, transported as dehydroascorbic acid, and reduced intracellularly to ascorbate [32]. Many cell types transport ascorbate only in its oxidized form, through facilitated glucose transporters [33]. Tumor cells have an increased requirement for glucose [34] and to compensate for this they increase the expression of glucose transporters. This allows ascorbate to act as a selective, non toxic to normal cells, chemotherapeutic.

## Conclusion

This study demonstrates that sodium ascorbate is highly toxic to neuroblastoma cell lines and the specific mechanism of vitamin C -induced apoptosis is due to a perturbation of intracellular iron levels ensuing TfR-downregulation.

This work, together with others, supports the use of vitamin C as an anticancer agent; in particular we suggest exploring the use of high dose intravenous ascorbate in controlled studies. Besides, vitamin C is relatively tumor specific, non toxic and inexpensive, in contrast to many chemotherapeutic agents in use.

## Methods

### Reagents

Sodium ascorbate and ferric ammonium citrate (FAC) were purchased from Sigma (St. Louis, MO). Ascorbate was dissolved in PBS, pH 7.4, and prepared immediately before use.

Monoclonal antibody to transferrin receptor (TfR, CD71) was obtained from BD biosciences (San Jose, CA).

### Cells

To broadly cover the phenotypes exhibited by NB cell *in vitro*, we used five human NB cell lines: SH-SY5Y [35], HTLA-230 [36], LAN-5 [37], IMR32 [38] and GI-LI-N [39]. All cell lines were grown in Dulbecco's modified Eagle medium supplemented with 10% fetal bovine serum (GIBCO Milan Italy) and 50 UI/ml penicillin, 50 μg/ml streptomycin and 2 mM L-glutamine (all reagent from Sigma). Cells were cultured at 37°C in a humidified atmosphere of 5% CO_2 _in air. Most experiments were performed on SH-SY5Y and HTLA-230, unless otherwise specified.

### Trypan blue exclusion method

The effects of sodium ascorbate on cell death were determined by trypan blue exclusion method as described elsewhere. Briefly, cells were seeded in 6-well plates, and, after 48 h, treated in absence or presence with various concentrations of vitamin C for 24 hours. Then, cells were trypsinized and diluted in growth medium. The cells were then counted under a phase-contrast microscopy, in a Burker counting chamber, in the presence of trypan blue (Sigma) solution at a 1:5 ratio, cells: trypan, (v/v).

### Cell cycle analysis

HTLA-230, IMR-32, LAN-5 and GI-LI-N were seeded in six well plates and treated in the absence or presence of increasing concentration of sodium ascorbate (0.5 mM, 1 mM, 2 mM and 3 mM) for 6 or 24 hours. Cells were harvested and washed with cold PBS, centrifuged at 1200 rpm and stored overnight in ethanol at -20°C. The following day, cells were washed with cold PBS, resuspended in 0.1% NP40, 100 μg/ml RNase A, 35 μg/ml propidium iodide (PI) and incubated for 20 minutes at 37°C. The stained cells were analyzed for DNA content by flow cytometry in a FACScan (Becton Dickinson, San Jose, CA) equipped with a 15-mW argon ion laser at 448 nm.

### Measurement of intracellular iron levels

HTLA-230 cells (30 × 10^6^) were cultured for 24 hours in presence or absence of 1.5 or 2 mM sodium ascorbate. Cells were then washed with PBS and homogenized in lysis buffer (10 mmol/L Tris-HCl, 144 mmol/L NaCl, 0.5% NP-40, 0.5% SDS, 1 mmol/L Na_3_VO_4 _plus protease inhibitors). Iron concentration in cell lysates was performed with a Cobas Integra 800 system (Roche Diagnostics GmbH, Mannheim, Germany) as routinarily assessed on plasma or serum samples by an Iron Test (Roche Diagnostics); iron concentration was estimated by absorbance at 552 nm.

### Measurement of mitochondrial membrane potential

Mitochondrial membrane potential was detected with the MitoPT™ Mitochondrial Permeability Transition Kit (Alexis).

Loss of the mitochondrial permeability transient event (PT) provides an early indication of the initiation of cellular apoptosis. This process is typically defined as a collapse in the electrochemical gradient across the mitochondrial membrane, as measured by the change in the membrane potential (ΔΨ_m_). Loss of the mitochondrial ΔΨ, can be detected by a unique fluorescent cationic dye, 5, 5', 6, 6'-tetrachloro-1, 1', 3, 3'-tetraethyl-benzamidazolocarbocyanin iodide, commonly known as JC-1, incorporated into the MitoPT™ kit. The dye penetrates cells and healthy mitochondria. Once inside a healthy non-apoptotic cell, the reagent bearing a positive charge, enters the negatively charged mitochondria were it aggregates and fluoresces red. When the mitochondrial ΔΨ collapses in apoptotic cells, the reagent no longer accumulates inside the mitochondria. Instead, it is distributed throughout the cell. When dispersed in this manner, the MitoPT™ reagent assumes a monomeric form, which fluoresces green. Then, it is easily distinguishable between non-apoptotic red fluorescent and apoptotic green fluorescent cells [40,41].

HTLA-230 and SH-SY5Y cells (1 × 10^6 ^cell/ml) were pretreated, or not, for 3 hours with 70 μg/ml FAC and then treated with sodium ascorbate for 18 hours. Cells were washed and resuspended in 0.5 ml of 1× MitoPT and incubated at 37°C for 15 minutes. After incubation, cells underwent to multi-parameter analysis using a flow cytometer.

### Annexin assay

Apoptotic cells were detected by Annexin V staining using the Annexin V-FITC kit purchased from Pharmingen (San Diego, CA) following the instructions of the manufacturer.

Briefly, HTLA-230 treated with sodium ascorbate and with or without FAC were washed with cold PBS and then resuspended in 1× binding buffer at a concentration of 5 × 10^5 ^cell/ml.

195 μl of cell suspension was transferred to a tube, then 5 μl of Annexin V-FITC was added and cells were incubated at room temperature for 10 minutes in the dark with gentle vortexing. Cells were then washed and resuspended in 200 μl of 1× binding buffer and analyzed by flow citometry.

### Caspase activity assay

The FLICA Apoptosis Detection Kit (Alexis, San Diego, CA) was used to detect ascorbate-induced activation of caspases in neuroblastoma cells, according to the manufacturer instructions, by flow cytometry. The FAM-VAD-FMK reagent provided in the kit is a carboxyfluorescein (FAM) derivative of benzyloxycarbonyl valyl alanyl aspartic acid fluoromethyl ketone (zVAD-FMK), which is a potent inhibitor of caspase activity. It enters the cell and irreversibly binds to activated caspases (caspases 1, 2, 3, 4, 5, 6, 7, 8, and 9) allowing for direct detection of pan-caspases activity by green fluorescence in live cells [42]. Briefly, cells were detached by trypsinization and centrifuged at 400 × g for 5 minutes at room temperature. Cell supernatants were removed and the pellets resuspended in a buffer containing the pan-caspase-fluorescent probe. After 1 hour of incubation, samples were washed and analyzed by single color flow cytometry at 488 nm for fluorescein on the FL1 channel.

To assess which caspase was mainly involved in ascorbate-induced apoptosis, we used all the commercially available kits specific for single caspases, i.e., FAM-YVAD-FMK for caspase-1, FAM-VDVAD-FMK for caspase-2, FAM-DEVD-FMK for caspases-3&7, FAM-VEID-FMK for caspase 6, FAM-LETD-FMK for caspase 8, FAM-LEHD-FMK for caspase 9 and FAM-AEVD-FMK for caspase-10. All kits were obtained from Alexis (San Diego, CA).

## Competing interests

The author(s) declare that they have no competing interests.

## Authors' contributions

All the authors have contributed significantly to the work.
